# Billroth

**Published:** 1877-04

**Authors:** 


Billroth.—A correspondent of the *New York Medical Record* draws this picture of Prof. Billroth: "A profound pathologist, an accurate anatomist, an operator bold to the verge of rashness, an easy conversational lecturer, an accomplished linguist, a good blackboard draughtsman, are qualities not every day to be found combined in one who during the most sever and tedious operations preserves an amiability and unpretentiousness which makes his presence a companionship to the youngest assistant. Nor does one often find the strength and endurance of a blacksmith uniting these qualities on one hand to a distinguished social reputation as a composer and pianist, on the other. A combination of qualities like this in one so favorably circumstanced could hardly fail in achieving the poplarity and success which Professor Billroth has accomplished.—*Louisville Medical News.*

---

The value of blue glass as a histiological forcer was nowhere better shown than in the case of the man who attempted to cure a wart on his nose by its use. In two weeks' time he was unable to tell which was the nose, and which was the wart.—*Med. News.*

